# Flanking p10 contribution and sequence bias in matrix based epitope prediction: revisiting the assumption of independent binding pockets

**DOI:** 10.1186/1472-6807-8-44

**Published:** 2008-10-16

**Authors:** Christian S Parry

**Affiliations:** 1 Computational Biophysics Section, Laboratory of Computational Biology, National Heart, Lung and Blood Institute, National Institutes of Health, Bethesda, Maryland 20892-9314, USA

## Abstract

**Background:**

Eluted natural peptides from major histocompatibility molecules show patterns of conserved residues. Crystallographic structures show that the bound peptide in class II major histocompatibility complex adopts a near uniform polyproline II-like conformation. This way allele-specific favoured residues are able to anchor into pockets in the binding groove leaving other peptide side chains exposed for recognition by T cells. The anchor residues form a motif. This sequence pattern can be used to screen large sequences for potential epitopes. Quantitative matrices extend the motif idea to include the contribution of non-anchor peptide residues. This report examines two new matrices that extend the binding register to incorporate the polymorphic p10 pocket of human leukocyte antigen DR1. Their performance is quantified against experimental binding measurements and against the canonical nine-residue register matrix.

**Results:**

One new matrix shows significant improvement over the base matrix; the other does not. The new matrices differ in the sequence of the peptide library.

**Conclusion:**

One of the extended quantitative matrices showed significant improvement in prediction over the original nine residue matrix and over the other extended matrix. Proline in the sequence of the peptide library of the better performing matrix presumably stabilizes the peptide conformation through neighbour interactions. Such interactions may influence epitope prediction in this test of quantitative matrices. This calls into question the assumption of the independent contribution of individual binding pockets.

## Background

It is essential to understand the host immune response in order to boost or modulate the immune system in infectious diseases, autoimmune diseases, allergies or cancer. This requires knowledge of the peptides selected and presented by class II major histocompatibility complex (MHC) molecules and the rules governing their binding and presentation to CD4+ T cells. Molecules of the MHC are surface receptors on immune cells that bind and present selected antigen as short peptides or epitopes to T cells with matching receptors. The peptides are produced by the proteolytic machinery of the antigen presenting cell. Class I epitopes are generated from intracellular proteins [1] and class II epitopes are processed from vesicular, endocytosed and cytosolic proteins through the exogenous pathway [2]. Processed peptides are loaded in intracellular compartments and transported to the cell surface where they are displayed for recognition by T cells. The unique design of the peptide binding region of the MHC and the vast polymorphism, through duplication, gene conversion and other genetic mechanisms, combine to generate hundreds of molecular variants at class I *HLA-A*, -*B*, -*C *and class II *HLA-D *loci [3]. Through these mechanisms MHC molecules are able to recognize and bind a vast array of peptides with fine distinction. Each allele has a different peptide binding specificity.

The MHC receptor comprises a membrane distal peptide binding domain sitting on a scaffold or platform formed by two immunoglobulin domains juxtaposed in a characteristic fashion. The peptide binding domain consists of eight anti-parallel beta strands on top of which lie two anti-parallel alpha helices. This is a unique fold and the two alpha helices form the walls of the peptide binding cleft [4]. Polymorphic residues in the beta sheet floor and in the alpha helical walls of the MHC form pockets that enforce genetic restriction and allele specificity [5-7]. Peptides that bind to class I molecules have a restricted length, about eight to eleven residues. The bound peptide forms hydrogen bonds with conserved residues at either end of the cleft effectively sealing them. Class II molecules, on the other hand, are open at either end and allow peptides of nearly unrestricted length to extend over the termini of the binding groove.

The register of the peptide cleft or binding groove in both class I and class II MHC molecules is nine residues [8]. The positions are labelled p1, p2, ..., p9, relative to the large N-terminal pocket in class II. Pockets p1, p4, p6 and p9 are prominent pockets in class II molecules; p3 and p7 are shallow shelves or minor pockets. Bound peptides in class II molecules adopt a polyproline type II-like conformation [9]. This near helical conformation allows the bound peptide to engage the major polymorphic pockets with anchor residues lodged in p1, p4, p6 and p9 leaving peptide side chains at p2, p3, p5, p7 and p8 simultaneously available for inspection by T cells. Peptide binding energy derives from the engagement of the peptide anchor residues in the MHC binding pockets with additional contribution from hydrogen bonds from the peptide backbone to conserved residues within the class II MHC molecule.

Methods to identify peptides that are immunogenic are important in basic and applied research – for fundamental understanding and for designing new drugs and vaccines to treat disease. Traditionally, this has meant synthesizing overlapping peptides covering the entire sequence followed by purification and direct or indirect assays of peptide binding to MHC molecules. This is time consuming and expensive. Reliable computational screening followed by experimental validation provides a more rapid and less expensive alternative. This may be carried out for several alleles to cover a wide segment of the population.

The restricting MHC molecule imposes structural constraints on the peptide anchors through polymorphic residues within the pockets [6,7]. This defines the *peptide binding motif *[10,11] for an allele. Related alleles have overlapping peptide repertoire and share a similar motif or core sequence [12,13]. Anchor motifs such as obtained through eluted natural ligands and phage display libraries have been useful in epitope prediction [12,14,15]. Quantitative matrices are extended motifs. They are an improvement over simple motifs and provide more informational content. Their coefficients describe the likelihood of an amino acid at a given location in a peptide to contribute to binding to an MHC allele [16-18]. They give high specificity, are fast, intuitive and easy to use.

Quantitative matrices are typically 20 × 9 in dimension reflecting the twenty natural amino acids and the nine residue register of the binding cleft of MHC molecules [16-18] as has been determined by X-ray crystallography [8]. The overall binding energy of the peptide is assumed to be the linear sum of the contribution of individual side chains. The peptide backbone hydrogen bonding contribution is ignored. This scheme is the independent binding of side chains approximation [16]. Equivalent matrices have been derived from the relative abundance of an amino acid at a given position from the sequence alignment profile of a library of known binders to an MHC allele [19]. Epitope prediction by quantitative matrices has been useful in identifying antigens in allergens [20], infectious agents such as Mycobacterium tuberculosis [21], and tumours [22]. Reviews by Korber and colleagues and by Tsurui and Takahashi give a current survey of the field [23,24].

Most prediction programs, whether they use quantitative matrices or machine learning methods, employ the canonical nine pocket binding register. There is growing appreciation that flanking residues influence peptide binding [25]. Flanking residues at the C-terminus of the binding groove of class II MHC molecules are polymorphic. The shelf formed at flanking position p10 makes potential sequence dependent contribution toward peptide binding energy. The equivalent flanking position at the N-terminus of the peptide groove is conserved; therefore the contribution of the position preceding pocket p1 (p-1) is ignored.

Results from structural and binding studies using the peptide AWCSDEALPLGSPRCD in complex with HLA-DRB3*0101 (HLA-DR52a) show a contribution from position p10. 24-AWCSDEALPLGSPRCD-39, from the N-terminus of human integrin α2βIIIa is a major ligand of DR52a. A natural variant of the epitope widely distributed in the population is 24-AWCSDEALP*P*GSPRCD-39. The Leu33/Pro33 dimorphism is the basis of the unidirectional alloimmune posttransfusion thrombocytopenia purpura (PTP) and fetal-maternal alloimmune thrombocytopenia (FMAIT), severe blood disorders in some DR52a subjects homozygous in Pro33 [26-28]. While AWCSDEALPP**G**S, or the second epitope 24-AWCSDEALPPGSPRCD-39 (Pro33), does not bind to DR52a AWCSDEALPP**L**S (leucine replacing glycine at position 10) has been demonstrated to bind. This has been confirmed by crystallographic studies (CSP, unpublished). These point to the contribution of leucine as an auxiliary anchor and the importance of position p10. Other studies of peptide-class II MHC complexes show a sequence dependent role for p10 [29].

In this study, we examine whether adding the contribution of position p10 improves prediction accuracy over a common 9-pocket matrix [17]. We construct two new quantitative scoring matrices for HLA-DR1 extended to include the contribution of flanking position p10. Coefficients for position 10 were constructed from the peptide library AA**Y**SD**Q**A**T**PL**L**XSPR, where X at position p10 is one of the twenty natural amino acids. Canonical anchor residues are in bold. The base peptide was derived from the N-terminal human integrin α2βIIIa peptide used for DR52a studies and has been designed to bind in a specific frame to HLA-DR1 by substituting well known anchor residues from the DR1 peptide binding motif and crystallographic studies [30,31]. A second peptide library was based on AA**Y**SD**Q**A**T**LL**L**XSPR where a proline has been replaced with leucine in order to avoid conformational effects due to proline. We find that the new matrix based on AAYSDQATPLLXSPR (called *PP10*, from the role of position 10 and the extra proline) shows significant improvement over the original nine residue register matrix, *P9*. The matrix based on AAYSDQATLLLXSPR (*P10*, from the role of position 10) does not show such improvement. The peptide libraries for the extended matrices differ in sequence at one position: proline versus leucine.

## Results

### Bioinformatic analysis

We have used IC_50 _values, obtained from binding assays of a variety of test peptides to DR1, to construct coefficients to extend the Hammer 9-register matrix [17] (*P9*) to position 10 of the peptide binding cleft. The protocol is described under **Methods**. The new extended matrices, *P10 *and *PP10*, are validated in regression analysis of predicted values against experimental binding measurements of peptides from glutamic acid decarboxylase (GAD65), islet cell antigens (ICA69) [32] and Varicella-zoster virus (VZV) (unpublished data). We compare these with results using the canonical 9-register matrix for DR1 [17]. The TEPITOPE virtual matrices for DR1 and other alleles [33] are based on this published matrix according to similarity in the pocket sequence profile. Logarithm of IC_50 _values are plotted against predicted values and a function is fit to the data. A fitting function is chosen whose coefficients minimize the residuals.

**Figure 1:**
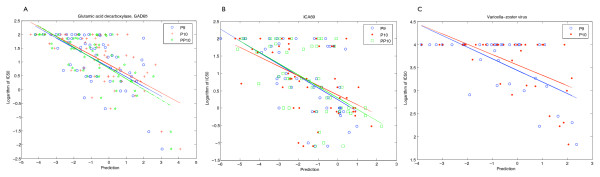
**Plots of binding measurements versus prediction values.** Half maximal inhibitory concentration (IC50) values of peptide sequences are plotted as a function of their predicted values for each of the three matrices, *P9 *(open circles, blue), *P10 *(cross, red) and *PP10 *(stars, green). A line is fitted to the plotted values in the respective colors. This is done for data sets A. Glutamic acid decarboxylase, GAD65; B. Islet cell antigen protein, ICA69; and C. Varicella zoster virus, VZV.

**Figure 2:**
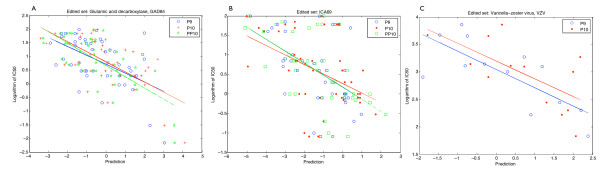
**Plots of edited binding measurements versus prediction values**. Binding measurements are again plotted against predicted values. Poor binders have been removed, and half maximal inhibitory concentration (IC50) values of peptide sequences are plotted for each of the three matrices, *P9 *(open circles, blue), *P10 *(cross, red) and *PP10 *(stars, green). A line is fitted to the plotted values in the respective colors for the respective data sets, A. Glutamic acid decarbolase, GAD65; B. Islet cell antigen, ICA69, and C. Varicella zoster virus, VZV.

The best fit was found to be a straight line. Higher order polynomial functions were tried but gave worse results. Low IC_50 _values correspond to good binding and high IC_50 _values correspond to poor binding, and negative predicted values. A flat fit has no predictive use. Plots for GAD65, ICA69 and VZV are shown in Figures 1 and 2. In all three scoring methods, there are both false positives (upper right quadrant) and false negatives (bottom left quadrant, Figures 1 and 2). False positives can be screened out in validation tests but false negatives are problematic. With these data sets, the scoring matrices show few false negatives. This is a useful property and the matrices can be used to screen large sequences without missing potential epitopes. A high threshold may be set to eliminate falsely predicted peptides.

In all three data sets, GAD65, ICA69 and VZV, for the three matrices tested there is correlation between binding (measured IC_50_) and predicted values (Figure 1). This indicates predictive value in all three matrices, the two new extended basis matrices *P10 *and *PP10 *and the control matrix *P9*. For GAD65 (Figure 1a), matrices of the original nine-residue matrix (*P9*) and the extended matrices (*P10 *and *PP10*) perform about equally from the fits. Using the slope *m *as a surrogate measure, *PP10 *is better than *P9 *(0.383 vs. 0.352, in absolute values) and *P9 *is in turn better than matrix *P10 *(0.352 vs. 0.341). The analysis is summarized in Table 1. For ICA69 (Figure 1b), *PP10 *and *P9 *have nearly the same slope (0.327 vs. 0.338) and better predictive power than *P10 *(0.275). The difference between the respective slopes of *P9 *and *PP10 *is not significant but the deviation between 0.327 and 0.338 (*PP10 *and *P9*) and 0.275 for *P10 *appears significant. Using the coarse criterion of slope *m *for VZV (Figure 1c), *P9 *is better than *P10 *(0.252 vs. 0.225). Visual inspection of the graphs supports these assertions (Figure 1). Interestingly, from Table 1, the difference in the absolute slope values between *P9 *and *P10 *for GAD65 and ICA69 data sets is 0.011. Difference in peptide-HLA-DR binding assay sensitivity between different antigenic proteins, data sets, individual experiments and the nature of competition assays makes it difficult to establish absolute numbers, and comparison between different data sets is difficult. Comparison within a data set is more valid as revealed by the plots.

**Table 1:** Summary of analysis

A. Full data				
Data set	method	-*m*	*J*	*r*^2^

GAD65	*P9*	0.352	30.22	0.373
	*P10*	0.341	26.75	0.445
	*PP10*	0.383	22.37	0.536
ICA69	*P9*	0.338	29.91	0.325
	*P10*	0.275	33.24	0.250
	*PP10*	0.327	29.27	0.340
VZV	*P9*	0.252	8.16	0.386
	*P10*	0.225	8.99	0.324

B. Data edited to remove poor binders				

Data set	method	-*m*	*J*	*r*^2^

GAD65	*P9*	0.324	25.44	0.279
	*P10*	0.368	20.12	0.429
	*PP10*	0.410	16.12	0.543
ICA69	*P9*	0.308	24.27	0.251
	*P10*	0.244	25.47	0.214
	*PP10*	0.308	21.22	0.345
VZV	*P9*	0.334	1.92	0.573
	*P10*	0.324	2.56	0.431

The fit of prediction to binding has been calculated for each prediction method using measures of slope (-*m*), sum of the square terms of the residual error (*J*) and *r*-square value (*r*^2^) for peptide sequences from GAD65, ICA69 and VZV. Table 1A shows the statistics for the unedited data, and Table 1B shows the equivalent statistics for the data edited to remove poor binding measurements.

Figures 1a, b and 1c show that low binding peptides (IC_50 _= 100 uM for GAD65 and ICA69 and 10 uM for VZV) are poorly predicted. This marks the limit of sensitivity of the experimental measurement. The analysis is repeated without these points: Figures 2a, b and 2c show the same plots when data points representing poor IC_50 _values are ignored. It is not clear how significant the difference between *P9 *and *P10 *slopes is for GAD65. Nevertheless, the conclusion that *PP10 *is superior is not changed. The VZV data set further reveals the difference in performance between *P9 *and *P10*. The magnitude of the slope used this way calibrates closely the "structure" or "informational potential" within the data; a flat fit signifies random data and has neither "structure" nor predictive potential.

**Figure 3:**
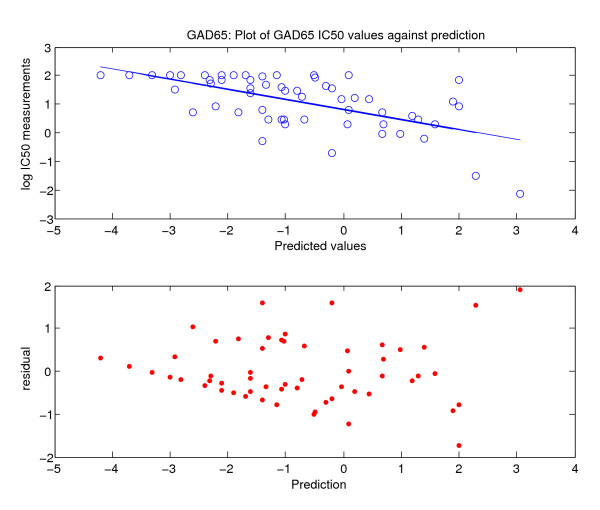
**Distribution of residuals for GAD65**. Values of half maximal inhibitory concentration are plotted for glutamic acid decarboxylase (GAD65) together with calculated residuals. The top panel is the plot of IC50 against prediction for GAD65; the bottom panel shows the distribution of the residuals.

The predictive potential is more accurately captured in terms of variance ("bandwidth") and residuals ("noise"). Residuals for the three data sets were calculated. A typical plot is shown in Figure 3 for GAD65 data set. The sum of the square of the residual values is calculated for each prediction matrix *P9*, *P10 *or *PP10*. Finally, the quality of the fit is assessed through the *r-*square value:

(1) $$r^{2}=\left(1-\frac{J}{S}\right)$$

*J *is the sum of the square terms of the residual error and *S *is the variance of the data. The analyses incorporating the slope *m *of the fitting function, residuals *J*, and *r*^2 ^are shown in Table 1. *r*^2 ^values range from 0.25 to 0.54 for the full data and 0.21 to 0.57 for the pruned set. The corresponding range for the magnitude of the slope is 0.22 to 0.38 for the full set and 0.24 to 0.41 for the edited set. The slopes shadow closely the respective *r*^2 ^values (Table 1).

From *r*^2 ^values, prediction method *PP10 *again works the best in both GAD65 and ICA69 data sets: *PP10 *performs better than the control matrix *P9 *and is superior to the other extended basis matrix *P10*. *P10 *is better than the control *P9 *matrix in GAD65 (*r*^2 ^of 0.45 versus 0.37) but not in ICA69 (0.25 versus 0.33). The ordering and conclusions are unchanged with filtered data. *PP10 *matrix has superior discrimination potential in terms of the slope *m *and also in terms of *r*^2^. *PP10 *shows marked improvement over matrix *P9 *and *PP10 *is also better than *P10*. While extended matrix *P10 *makes better prediction than *P9 *in GAD65 it is worse than *P9 *in the edited ICA69 data set. In VZV, matrix *P10 *shows no improvement over *P9 *in either the filtered or unfiltered data sets, according to *r*^2^. Overall, average *r*^2 ^values of the raw data sets (Table 1) are 0.36, 0.34 and 0.44 respectively for *P9*, *P10 *and *PP10*. For comparison, reported average *r*^2 ^value of a large-scale evaluation of class II molecules using similar quantitative matrices is 0.25[34].

### Structural analysis

To gain structural insight into epitope prediction, and why matrix *PP10 *performs better than *P9*, we examined peptides that bind well according to IC_50 _binding measurements. The best experimental binding measurement is for peptide 54 from GAD65 (GDKVNFFRMVISNPAATHQD, *p*IC_50 _= 2.15; Supplementary table, Additional file 1). The same register FRMVISNPA(A) is predicted as a strong binder for both matrices, *P9 *(predicted score = 3.05) and *PP10 *(predicted score = 3.58). Anchor residues at dominant pockets p1, p4, p6 and p9 are as expected for DR1 [12]. Ala at position p10 is also predicted to contribute to favourable binding. Incidentally, matrix *P10 *is in agreement: the same binding frame is predicted and Ala at the extended position is also predicted to interact with the shallow shelf at position p10 for enhanced binding. The added contribution of Ala is given greater weight in matrix *P10 *(1.05 units) than in *PP10 *(0.53 units). Competition binding assay of the peptides used for the libraries shows that Ala is a preferred residue at p10 for HLA-DR1.

A unanimous binding frame (in bold letters) is also predicted from peptide 9 (Supplementary table, Additional file 1) SCSKVDVN**YAFLHATDL**LPA, another strong binder. Leucine at position p10 is predicted to contribute to greater binding affinity, in agreement with experiment. Matrices *P9 *and *PP10 *predict the same frame (in bold letters) in peptide 4, QVAQK**FTGGIGNKL**CALLYG (Supplementary table, Additional file 1). Strong peptide anchor residues at p1 and p9 (F and L, respectively) compensate for poor anchors (G) at p4 and p6, and the contribution of cysteine at position p10 is predicted to enhance binding from matrix *PP10*. The predicted binding frame in matrix *P10*, however, differs – IGNKLCALLY.

Another example shows how the extended position changes the predicted binding frame between matrices *P9 *and *PP10*: in SHFSLKKGAAALGIGTDSVI (peptide 28), LKKGAAALG is predicted to bind best by matrix *P9 *while FSLKKGAAAL is predicted by the two extended basis matrices. LKKGAAALG (predicted by *P9*) has a good leucine anchor at p1 but three poor anchors at p4, p6 and p9. For matrices *P10 *and *PP10*, a strong phenylalanine anchor at position p1 in the peptide predicted combines with lysine at p4 and the auxiliary leucine anchor at position 10 to improve the score.

The peptide VETFRHRAISDTWLTVNRME from ICA69 is another example where the extended matrix *PP10 *predicts a frame (FRHRAISDTW, score = 0.27) different from that predicted by *P9 *(VETFRHRAI, score = -1.15). The better score predicted by *PP10 *may arise from an aromatic hydrophobic anchor in pocket 1 versus a relatively small aliphatic p1 anchor, valine, predicted by matrix *P9*. In the latter, phenylalanine is disfavoured in pocket 4, and likewise histidine in pocket 6, in the frame predicted by matrix *P9*. The other extended matrix *P10 *predicts the same frame as *PP10 *but with a much worse score (-1.75). This implies a predicted negative contribution of tryptophan at position p10 for matrix *P10 *but a positive contribution of tryptophan for *PP10*. Experimental binding measurements support a positive contribution of tryptophan at pocket 10. The binding frame predicted by matrix *PP10 *is structurally consistent with the measured binding affinity.

In peptide 117, the ICA69 peptide QCRTEYRGALLWMKDVSQEL (Supplementary table, Additional file 1) is predicted to bind in a different frame (CRTEYRGAL, score = -0.52) than that predicted for *PP10 *(YRGALLWMKD, score = -1.15). Non-ideal anchors (Cys at p1 and Glu at p4) predicted by matrix *P9 *are compensated by good anchors (Arg at p6 and Leu at p9). The frame predicted by *PP10 *possesses a large and favourable hydrophobic anchor (Tyr) at p1, a favoured small residue (Ala) at p4 but Leu and Lys at pockets p6 and p9, respectively. Leu and Lys are disfavoured in these positions. The contribution at position p10 does not overcome these hindrances.

AIHESFKGYQPYEFTTLKSL and RKESSSFKTEDGKSILSALD (peptides 126 and 132; Supplementary table, Additional file 1) are predicted as strong binders, in agreement with experimental data, and in the expected registers, FKGYQPYEF(T) and FKTEDGKSI(L), for all three matrices. In peptide 126, polar threonine is not favoured at position p10; this reduces the score slightly in both extended matrices. On the other hand, in peptide 132, hydrophobic leucine anchor at p10 in the predicted fragment enhances the interaction between DR1 and the peptide ligand to give a slightly higher score for both extended matrices *P10 *and *PP10*.

And in another example, the predicted register is shifted one position between matrices *P9 *and *PP10 *for peptide SVVNKMQQRYWETKQAFIKA (peptide 103; Supplementary table, Additional file 1): **V**NK**M**Q**Q**RY**W **and **V**VN**K**M**Q**QR**Y**W (p1, p4, p6 and p9 anchors are in bold). The added basis in *PP10*, the increased number of parameters, has allowed better prediction accuracy.

Finally, all three matrices unanimously select the same best frame YGAFDPLLA(V) from overlapping peptides 33 and 34 (Supplementary table, Additional file 1). The experimental *p*IC50 values are 0.05 (peptide 33) and -0.70 (peptide 34) compared to predicted scores of 0.68, 0.33 and 0.45 for *P9*, *P10 *and *PP10*, respectively. Similarly, the same frame FRKVQTQVRL is unanimously selected by the three matrices for peptides 119 and 120 (Supplementary table, Additional file 1). *p*IC50 values are 0.22 (peptide 119) and 0.00 (peptide 120). Predicted scores are 0.15, 0.90 and 1.42 for *P9*, *P10 *and *PP10*, respectively, for the unanimously predicted best frame for the two peptides. In the former pair of peptides, the experimental values are far apart while the computational scores are relatively close. In the latter set, the experimental values are close but the predicted scores show a big spread.

Statistics for predicted scores are easy to obtain but similar statistics for binding measurements are difficult to obtain as little statistical data is given along with the experimental data. As is typical of such measurements, baseline values are determined on a case by case basis.

## Discussion

An important contribution to the binding affinity of peptide to class II MHC is the aggregate of the interaction of individual peptide side chains with the polymorphic pockets. The contribution of backbone atoms is ignored as is formalized in the independent binding of pockets hypothesis [16] that underlies epitope prediction algorithms based on quantitative matrices. Methods of epitope prediction, including quantitative matrices, typically consider only the nine positions or pockets within the binding groove. There are reports that show, however, that sequences flanking the binding cleft affect peptide binding affinity [25]. This contribution when accounted for may therefore improve epitope prediction accuracy for class II MHC alleles. The termini of the binding groove of class I molecules are sealed.

We constructed two matrices *P10 *and *PP10 *that incorporate flanking position p10 and tested them against experimental binding data. Extended matrix *PP10 *showed significant improvement over the canonical 9-mer matrix, *P9*. In validation tests of two typical data sets extended matrix *PP10 *showed superior performance (average *r*^2 ^= 0.440) over the original *P9 *matrix (average *r*^2 ^= 0.350). On the same test sets, extended matrix *P10 *performed no better (average *r*^2 ^= 0.350) than *P9 *(Table 1).

The superior performance of prediction matrix *PP10 *is best explained in terms of additional position p10 in the binding register. This explicitly increases the number of parameters. A major conclusion therefore is that incorporating the flanking position may improve class II MHC epitope prediction. Extended matrix *PP10 *also selects a more plausible binding register than what is predicted by matrix *P9*. The relative poor performance of matrix *P10 *is best explained in terms of the difference in sequence of the peptide library it was based on. It is not clear the effect of the -LLL- subsequence (in *P10*) on the peptide conformation. Conversely, it is conceivable that the better performance of matrix *PP10 *derives from greater stabilization of the peptide polyproline II-like conformation by the extra proline in the sequence of the peptide library.

The left handed polyproline II helical conformation is found in structural proteins, in signalling molecules and as ligands in MHC complexes [9]. Proline is unusual among natural amino acids. In a polypeptide, its φ torsion angle is restricted to -63°. The δ carbon of the proline ring interacts with the backbone N and constrains the preceding residue [35]. Such influence of proline on the adjacent residue is contrary to the assumption of independent binding of peptide side chains and needs accounting in quantitative scoring matrices.

Many lines of structural studies of peptide-MHC complexes suggest cooperative interactions between pockets [36]. Peptide selection and binding may be compared to sampling the conformational space available to the complex of peptide and MHC. The path followed and the final structure adopted by the complex depend on sequence, and these involve cooperative interactions. The accompanying conformational changes are not accounted for in quantitative matrices. A related issue is the stability of the bound complex.

Hydrogen bonds from the peptide backbone are a major contribution to peptide-class II MHC binding. Experimental structures show 12–16 such bonds from the peptide backbone to usually conserved residues in the class II molecule. Each such hydrogen bond is important for binding affinity and stability [37]. While, in principle, these backbone hydrogen bonds enable peptide binding regardless of sequence the number of such bonds may vary depending on the peptide sequence. Proline residues in a peptide sequence can alter the pattern and number of such backbone hydrogen bonds. Neither quantitative matrices nor the assumption of independent binding takes backbone hydrogen bonding into account.

Further, a given peptide-class II complex is best represented as a heterogeneous set of conformations of varying stability. These conformational dynamics are a function of the particular peptide in the MHC and reflect sequence, near neighbour and long range pocket interactions. This is another limiting factor in epitope prediction. Already, sequence dependence is assumed in functional work and sequence sensitivity to selectivity and stability of class II ligands has been demonstrated in the catalysis of peptide loading down to the level of hydrogen bonds [36,38,39].

Peters and colleagues have proposed the method of stabilized matrices [40] to account for near neighbour and cooperative interactions between pockets. Their calculated coefficients are used to augment the well known quantitative matrices used in TEPITOPE [33]. The added terms are small, about a factor of 10 smaller than the entries of the original quantitative matrices. However, stabilized matrices improved prediction performance over the original quantitative matrix, and to a level equalling or exceeding that of general (machine learning) methods.

This report does not address questions about peptides that are poorly predicted. Experimental limitations are a factor in poor predictions but are difficult to quantify. Measured IC_50 _values are relative to a reference peptide, and sensitivity may vary from assay to assay. It is also not always clear what is binding. If bound peptide is what is being measured, other questions arise such as in what frame. An examination of crystallographic structures of peptide-class II MHC complexes shows that peptide anchors are not always fully engaged in binding pockets especially after pocket p6; in the collagen peptide complex with DRB5*0101 [41] the peptide side chain at p9 is out of pocket and extends rather to the shelf at position 10. In general, not all pockets need to be fully engaged for good peptide binding [42]. Short of experimentally derived structures exactly how peptides bind in each complex, or whether they bind at all in the binding site, as measured in assays, has to be assumed. These issues are intimately linked with the biology of the system.

The biology and the experimental restriction of the system are important limiting factors in T cell epitope prediction accuracy. Immunoassays need to become reliably quantitative in order to ensure accuracy in prediction. Heterogeneity of peptide conformations also needs to be addressed. These are difficult issues but they must be taken into account in interpreting results from epitope prediction.

## Conclusion

This study set out to find whether the contribution of flanking pocket 10 improves prediction accuracy. Two matrices incorporating position p10 were constructed. Tests showed that the contribution of the added basis describing position p10 may lead to improved prediction over the usual nine residue register matrix. The added dimension explicitly increases the parameters. The study also revealed the importance of sequence context.

The aim of modern biology is to explain physiological phenomena not only at the level of cells and tissues but also at the molecular level. This requires full knowledge of the coding sequence and the atomic structure of the molecules. Nevertheless, the three dimensional representation of molecules of itself is inadequate. Near neighbour interactions between residues, long range cooperative effects between domains and other tempo-spatial effects are just as important, hence a need for representations that capture these.

Quantitative matrices used in epitope prediction are actually an example of such parameterization. A matrix coefficient contains parameterization of the physical chemical properties of pocket shape, peptide side chain to pocket interactions and electrostatics without explicit description. Sequence dependence is considered on the basis of individual pockets. The approach followed here similarly aims at increasing the parameters of the scoring matrix by explicitly increasing the basis through flanking position p10. The method of stabilized matrices has demonstrated a way of introducing small additional terms describing cooperative effects [40].

Comparing the performance of the two extended matrices described here has made evident implicit sequence bias in the matrices. Proper accounting of sequence effect will further contribute to prediction accuracy. Peptide libraries are implicitly optimized through the incorporation of favourable residues at anchor positions; these capture salient nonlinear features of peptide conformations. Fuller understanding may lead to a judicious choice of basis, parameters or peptide library to construct scoring matrices that capture more of these cooperative interactions to improve prediction accuracy.

## Methods

### Generating coefficients

Matrix coefficients to extend the binding register to position p10 are derived following the method described previously [17]. A designed peptide carrying the test amino acid residue at position p10 is used in competitive human leukocyte antigen binding assays with purified DR1 allele. The peptide library was designed after the human integrin β3 peptide AWCSDEALPLGSPRC, a natural ligand of HLA-DR52a (DRA/DRB3*0101), and altered to incorporate anchor residues favourable to HLA-DR1, Y, Q, T and L, as deduced from the known peptide binding motif [30]. The first series called *PP10 *is AA**Y**SD**Q**A**T**PL**L**XSPR; anchor residues, at positions 1, 4, 6 and 9 (p1, p4, p6 and p9) in bold, and X represents one of the twenty natural L amino acids. In order to obtain coefficients for a residue X at p10, the amino acid at that position within the peptide is varied and the IC_50 _value is measured (described below). The IC_50 _value is normalized with the IC_50 _value of the Ala-substitution at position p10 for each residue X. The logarithm of the reciprocal value of normalization is obtained as:

$$\log\left(\frac{IC50_{Ala}}{IC50_{X}}\right)$$

Another peptide library is constructed based on the peptide AA**Y**SD**Q**A**T**LL**L**XSPR, *P10*. Proline has been replaced with leucine to avoid the backbone restriction and conformational heterogeneity of the pyrrolidine ring that are associated with proline. The extended matrix coefficients are calculated independently for pocket 10 with no assumption of neighbouring pockets or peptide positions. The free energy of binding of a peptide is approximated by the sum of individual side chain contribution within the binding register, the Independent Binding of Side Chains [16].

### Peptide binding experiments

The affinity of test peptides of the peptide library to HLA-DR1 was determined in standard competition binding experiments. 25 nM of the unliganded class II molecule was incubated with an equimolar amount of the probe, biotinylated influenza virus HA [306–318] peptide and unlabelled competitor peptide; the amount of the competitor peptide used is varied for each assay. The assay mixture is incubated in 50 mM NaCl, 100 mM Na_3_PO_4_, pH = 5.5, a cocktail giving final concentration 1 mg/ml PMSF, 37 ug/ml iodoacetamide, 10 mM EDTA, 0.02% NaN_3 _and 0.5 mg/ml octylglucoside at 37°C until equilibrium is reached after 3 days. This was followed by immunoassay using the anti-HLA-DR1 antibody LB3.1 in streptavidin to detect the bound biotinylated HA [306–318] peptide. IC_50 _values are deduced for each peptide from fluorescence values with respect to a range of concentration of the competitor peptide [31].

### Test set

The performance of the extended matrices was evaluated in prediction against experimental binding measurements of peptides derived from 65 kDa glutamic acid decarboxylase (GAD65) [32], 69 kDa islet cell antigens (ICA69) [32] and Varicella-zoster virus (VZV) (unpublished data). GAD65 and ICA69 data consist of 20-mer peptides overlapping by 10 amino acids and covering the complete sequence of the proteins. VZV peptides contain a variety of 15-mer through 27-mer peptides also overlapping by 10 amino acids and covering the entire range of the sequence.

### Validation

For each data set, a dependent variable was derived from experimental IC_50 _values through a logarithmic transform. This is correlated with predicted peptide scores through a fitted function by regression. Linear and nonlinear functions are used and evaluated based on the residual. The predicted scores are further evaluated through the *r*^2 ^(R-square) function. Regression analysis was carried out using MATLAB . Predictions were carried out within the in-house MHC epitope prediction tool PREDICT (CSP, unpublished).

## Authors' contributions

CSP wrote the prediction platform, tested and installed it. He programmed further for the extended matrices project; carried out the analysis; wrote and edited the manuscript.

## Supplementary Material

Additional File 1 **Supplementary table** Click here for file
