# Supplementary material for: Flanking p10 contribution and sequence bias in matrix based epitope prediction: revisiting the assumption of independent binding pockets
Source: BMC Struct Biol. 2008 Oct 16;8:44. doi: 10.1186/1472-6807-8-44 (PMC2600787; doi:10.1186/1472-6807-8-44)
Supplement: Additional File 1 — Supplementary table [file 1472-6807-8-44-S1.doc]

## Supplementary table – Experimental binding affinity and predicted score of selected peptide

| peptide | sequence | *p*IC_50_exp | *P9* best frame | score | *P10* best frame | score | *PP10* best frame | score |
| --- | --- | --- | --- | --- | --- | --- | --- | --- |
| 1  4  9  12  13  14  18  19  21  22  23  24  25  26  27  28  32  33  34  36  48  49  52  54  103  104  106  107  108  109  110  111  112  115  117  119  120  122  123  124  125  126  127  132  133  135  136  138  139  141  143  144  145  146 | MASPGSGFWSFGSEDGSGDS  QVAQKFTGGIGNKLCALLYG  SCSKVDVNYAFLHATDLLPA  LQDVMNILLQYVVKSFDRST  YVVKSFDRSTKVIDFHYPNE  KVIDFHYPNELLQEYNWELA  IKTGHPRYFNQLSTGLDMVG  QLSTGLDMVGLAADWLTSTA  NTNMFTYEIAPVFVLLEYVT  LKKMREIIGWPGGSGDGIFS  PGGSGDGIFSPGGAISNMYA  PGGAISNMYAMMIARFKMFP  MMIARFKMFPEVKEKGMAAL  EVKEKGMAALPRLIAFTSEH  PRLIAFTSEHSHFSLKKGAA  SHFSLKKGAAALGIGTDSVI  EAKQKGFVPFLVSATAGTTV  LVSATAGTTVYGAFDPLLAV  YGAFDPLLAVADICKKYKIW  MHVDAAWGGGLLMSRKHKWK  LYNIIKNREGYEMVFDGKPQ  HTNVCFWYIPPSLRTLEDNE  PVIKARMMEYGTTMVSYQPL  GDKVNFFRMVISNPAATHQD  SVVNKMQQRYWETKQAFIKA  WETKQAFIKATGKKEDEHVV  ASDADLDAKLELFHSIQRTC  ELFHSIQRTCLDLSKAIVLY  LDLSKAIVLYQKRICFLSQE  QKRICFLSQEENELGKFLRS  ENELGKFLRSQGFQDKTRAG  QGFQDKTRAGKMMQATGKAL  KMMQATGKALCFSSQQRLAL  VETFRHRAISDTWLTVNRME  QCRTEYRGALLWMKDVSQEL  DPDLYKQMEKFRKVQTQVRL  FRKVQTQVRLAKKNFDKLKM  DVCQKVDLLGASRCNLLSHM  ASRCNLLSHMLATYQTTLLH  LATYQTTLLHFWEKTSHTMA  FWEKTSHTMAAIHESFKGYQ  AIHESFKGYQPYEFTTLKSL  PYEFTTLKSLQDPMKKLVEK  RKESSSFKTEDGKSILSALD  DGKSILSALDKGSTHTACSG  PIDELLDMKSEEGACLGPVA  EEGACLGPVAGTPEPEGADK  DDLLLLSEIFNASSLEEGEF  NASSLEEGEFSKEWAAVFGD  GQVKEPVPTMALGEPDPKAQ  TGSGFLPSQLLDQNMKDLQA  LDQNMKDLQASLQEPAKAAS  SLQEPAKAASDLTAWFSLFA  DLTAWFSLFADLDPLSNPDA | -0.70  0.70  1.52  -0.60  -0.77  -0.48  0.22  -0.30  -0.48  -0.48  -0.48  -0.30  -0.48  -0.78  -0.30  0.30  0.05  0.05  -0.70  -0.70  -0.90  -0.30  -0.90  2.15  1.00  0.10  -0.60  0.10  0.00  -0.30  -0.30  0.00  0.15  1.10  1.10  0.22  0.00  0.00  0.70  -1.00  0.00  0.10  -0.60  0.52  -0.90  -0.60  -0.60  0.10  -0.85  -0.85  -0.85  -0.85  -0.70  -0.78 | FWSFGSEDG  FTGGIGNKL  YAFLHATDL  MNILLQYVV  VVKSFDRST  LLQEYNWEL  YFNQLSTGL  MVGLAADWL  YEIAPVFVL  MREIIGWPG  FSPGGAISN  YAMMIARFK  FKMFPEVKE  LPRLIAFTS  FTSEHSHFS  LKKGAAALG  FVPFLVSAT  YGAFDPLLA  YGAFDPLLA  LLMSRKHKW  YNIIKNREG  CFWYIPPSL  MEYGTTMVS  FRMVISNPA  VNKMQQRYW  FIKATGKKE  LELFHSIQR  FHSIQRTCL  IVLYQKRIC  FLSQEENEL  FLRSQGFQD  MMQATGKAL  MMQATGKAL  VETFRHRAI  CRTEYRGAL  FRKVQTQVR  FRKVQTQVR  CQKVDLLGA  CNLLSHMLA  LLHFWEKTS  WEKTSHTMA  FKGYQPYEF  FTTLKSLQD  FKTEDGKSI  ILSALDKGS  MKSEEGACL  LGPVAGTPE  LLLLSEIFN  FSKEWAAVF  VKEPVPTMA  FLPSQLLDQ  MKDLQASLQ  LTAWFSLFA  FADLDPLSN | -1.82  -0.20  2.29  1.20  -1.41  -1.08  1.39  0.70  1.30  -0.68  -1.30  1.59  -1.02  0.10  -1.00  -1.40  0.98  0.68  0.68  -2.60  1.99  0.07  -2.21  3.05  -1.51  -1.30  -0.82  0.29  0.09  -0.50  -0.90  0.10  0.10  -1.15  -0.52  0.15  0.15  -0.55  0.59  -2.62  -3.01  0.10  -1.22  0.98  -3.00  0.10  -2.36  -1.10  -1.10  -3.11  -2.70  -2.10  -3.10  -1.50 | FWSFGSEDGS  IGNKLCALLY  YAFLHATDLL  MNILLQYVVK  VVKSFDRSTK  LLQEYNWELA  YFNQLSTGLD  MVGLAADWLT  YEIAPVFVLL  MREIIGWPGG  FSPGGAISNM  ISNMYAMMIA  FKMFPEVKEK  LPRLIAFTSE  FTSEHSHFSL  FSLKKGAAAL  FVPFLVSATA  YGAFDPLLAV  YGAFDPLLAV  LLMSRKHKWK  YNIIKNREGY  WYIPPSLRTL  MEYGTTMVSY  FRMVISNPAA  VNKMQQRYWE  FIKATGKKED  LELFHSIQRT  LDLSKAIVLY  LDLSKAIVLY  FLSQEENELG  FLRSQGFQDK  GKMMQATGKA  MMQATGKALC  FRHRAISDTW  YRGALLWMKD  FRKVQTQVRL  FRKVQTQVRL  LLGASRCNLL  LSHMLATYQT  YQTTLLHFWE  WEKTSHTMAA  FKGYQPYEFT  YEFTTLKSLQ  FKTEDGKSIL  ILSALDKGST  MKSEEGACLG  LGPVAGTPEP  LLLLSEIFNA  FSKEWAAVFG  VKEPVPTMAL  LLDQNMKDLQ  MKDLQASLQE  SLQEPAKAAS  FADLDPLSNP | -2.16  0.10  3.04  2.20  -1.41  -0.03  1.50  1.50  2.05  0.28  -0.82  1.84  -0.02  0.60  -0.25  -0.95  2.03  0.33  0.33  -1.60  2.69  0.75  -0.51  4.10  -2.01  -1.19  -0.02  0.80  0.80  0.46  0.10  -2.26  0.04  -1.75  -1.27  0.90  0.90  -0.35  -1.01  -2.52  -1.96  0.90  -1.55  1.73  -2.20  1.06  -2.82  -0.05  -0.14  -2.36  -2.45  -1.60  -4.94  -1.96 | FWSFGSEDGS  FTGGIGNKLC  YAFLHATDLL  MNILLQYVVK  VVKSFDRSTK  IDFHYPNELL  YFNQLSTGLD  MVGLAADWLT  YEIAPVFVLL  LKKMREIIGW  FSPGGAISNM  ISNMYAMMIA  FKMFPEVKEK  LPRLIAFTSE  FTSEHSHFSL  FSLKKGAAAL  FVPFLVSATA  YGAFDPLLAV  YGAFDPLLAV  VDAAWGGGLL  YNIIKNREGY  WYIPPSLRTL  MEYGTTMVSY  FRMVISNPAA  VVNKMQQRYW  FIKATGKKED  LELFHSIQRT  FHSIQRTCLD  VLYQKRICFL  FLSQEENELG  FLRSQGFQDK  GKMMQATGKA  MMQATGKALC  FRHRAISDTW  YRGALLWMKD  FRKVQTQVRL  FRKVQTQVRL  LLGASRCNLL  MLATYQTTLL  LLHFWEKTSH  WEKTSHTMAA  FKGYQPYEFT  FTTLKSLQDP  FKTEDGKSIL  ILSALDKGST  MKSEEGACLG  LGPVAGTPEP  LLLLSEIFNA  FSKEWAAVFG  VKEPVPTMAL  SGFLPSQLLD  LQASLQEPAK  AKAASDLTAW  FSLFADLDPL | -1.43  0.37  3.56  1.54  -2.07  -0.53  1.62  0.68  2.57  0.57  -1.22  1.32  -0.68  -0.52  0.27  -0.43  1.51  0.45  0.45  -1.93  0.91  1.27  -2.29  3.58  -0.73  -1.07  -0.84  0.52  0.57  -0.38  -0.56  -2.78  0.67  0.27  -1.15  1.42  1.42  0.17  -0.94  -2.79  -2.48  0.08  -1.31  2.25  -3.02  0.22  -2.45  -0.57  -0.98  -1.84  -2.47  -2.56  -4.03  -1.45 |

Measured and predicted values of peptides from GAD65 (labelled 1 through 100) and ICA69 (labelled 101 and above) after Geluk et al. [32] pruned to remove poor binders are listed below. These are examined to discern the structural basis of epitope prediction using the canonical 9-residue register (*P9*) and the extended register (*P10* and *PP10*) matrices. A suitable threshold value may be chosen to align measured *p*IC_50_exp with predicted score.
